# Comparison of Visual Functions of Two Amazonian Populations: Possible Consequences of Different Mercury Exposure

**DOI:** 10.3389/fnins.2019.01428

**Published:** 2020-01-21

**Authors:** Eliza Maria da Costa Brito Lacerda, Givago da Silva Souza, Maria Izabel Tentes Cortes, Anderson Raiol Rodrigues, Maria Conceição Nascimento Pinheiro, Luiz Carlos de Lima Silveira, Dora Fix Ventura

**Affiliations:** ^1^Faculdade de Biomedicina, Universidade CEUMA, São Luís, Brazil; ^2^Núcleo de Medicina Tropical, Universidade Federal do Pará, Belém, Brazil; ^3^Instituto de Ciências Biológicas, Universidade Federal do Pará, Belém, Brazil; ^4^Faculdade de Enfermagem, Universidade Federal do Amapá, Macapá, Brazil; ^5^Instituto de Psicologia, Universidade de São Paulo, São Paulo, Brazil

**Keywords:** neurotoxicology, mercury vapor, organic mercury, visual field, color vision, Amazon region, psychophysics

## Abstract

The present study investigated the visual perimetry and color vision of two Amazonian populations differently exposed to mercury. Ten riverines environmentally exposed to mercury by fish eating and 34 gold-miners occupationally exposed to mercury vapor. The visual perimetry was estimated using the Förster perimeter and the color vision was evaluated using a computerized version of Farnsworth–Munsell test. Riverine and gold-miners’ hair mercury concentrations were quantified. Mercury hair concentration of the riverines was significantly higher than that from gold-miners. Riverines had lower perimetric area than the gold-miners. The errors in the hue ordering test of both Amazonian populations were larger than the controls (non-exposed subjects), but there was no difference between themselves. Riverines had significant multiple association between the visual function and hair mercury concentration, while the gold-miners has no significant association with the exposure. We concluded that the different ways of mercury exposure led to similar visual outcomes, with greater impairment in riverines (organic mercury exposed subjects).

## Introduction

Since the end of the 1980s, there has been a growing concern with the environmental contamination of the Amazon region by mercury. The “gold rush” in the Amazon region brought about the unselective use of mercury in the process of mineral extraction, resulting in a large deposition of mercury in the Amazon rivers (Malm et al., 1995). This happens because of a high natural mercury concentration in the soil, to which deforestation, erosion, and spray of anthropogenic mercury contamination through rainfall are added (Roulet et al., 1998). Amazon rainforest soil retains mercury as a result of continuous input of mercury from the atmosphere and weathering processes on the rocks (Figueiredo et al., 2018). Deforestation can significatively increases the soil-atmosphere mercury exchanges and the leaching process that increases the mercury release from the soil to the rivers (Magarelli and Fostier, 2005).

Human exposure in the Amazon may occur in miners, directly by inhalation of metallic mercury vapor in the process of gold extraction through occupational exposure, or in riverines, indirectly by consumption of fish containing methylmercury accumulated in the food chain (Kershaw et al., 1980; WHO, International Program on Chemical Safety, 1989; Malm et al., 1995; Akagi and Naganuma, 2000). After the incidents of high mercury exposure in Minamata, Japan, the brain was the main target of the mercury toxicity (Amin-Zaki et al., 1976), mercury neurotoxicity involves neuronal destruction, beginning with effects on the occipital cortex and cerebellum (Takeuchi, 1968), and the clinical manifestations include loss of vision and hearing, mental disturbances, impairment of verbal learning and memory, paresthesia, ataxia, neurasthenia, spasticity, tremor, and reduction of concentration, tendon reflex, manual dexterity, fine motor speed and dexterity, salivation, and even coma and death (Takeuchi, 1977; Ekino et al., 2007).

Our knowledge about the visual consequences associated with mercury vapor exposure came from investigations with workers of factories that manipulated mercury in some stage of the productive process (Ventura et al., 2004, 2005; Feitosa-Santana et al., 2007, 2008). Few studies have reported repercussions of the occupational mercury exposure in Amazonian gold-miners that inhaled mercury vapor (Rodrigues et al., 2007; da Costa et al., 2008). Other studies have examined fish or seafood consumers to detect visual sequels associated with environmental exposure to mercury. Specifically, in the Brazilian Amazon region, most of the investigation has been done in riverside populations dependent on fishery (Lebel et al., 1996, 1998; Rodrigues et al., 2007; Lacerda, 1997; Passos and Mergler, 2008; Fillion et al., 2011, 2013; Dos Santos Freitas et al., 2018; Feitosa-Santana et al., 2018). The literature describes that mercury exposure is associated with several visual impairments – decreased color vision, reduced contrast sensitivity, visual field constriction (Lebel et al., 1996, 1998; Rodrigues et al., 2007; Fillion et al., 2011, 2013).

As the mercury biochemical pathways in the human body are different for mercury vapor and methylmercury from food, the comparison of visual functions between two populations with similar genetic background, but different exposure type could help to understand the visual function changes associated with mercury exposure. In the present study we compared the visual field perimetry and the hue ordering of Amazonian populations that had mercury exposure to methylmercury through fish consumption or to mercury vapor through occupational exposure.

## Materials and Methods

### Study and Ethics Statements

This is a cross-sectional descriptive and analytical observational study. All subjects gave written and informed consent to participate in this study. All procedures were evaluated and approved by the Ethics Committee in Research in Humans of the Tropical Medicine Center of the Federal University of Pará (Protocol #021/2009-CEP-NMT/UFPA).

### Population

**FIGURE 1:**
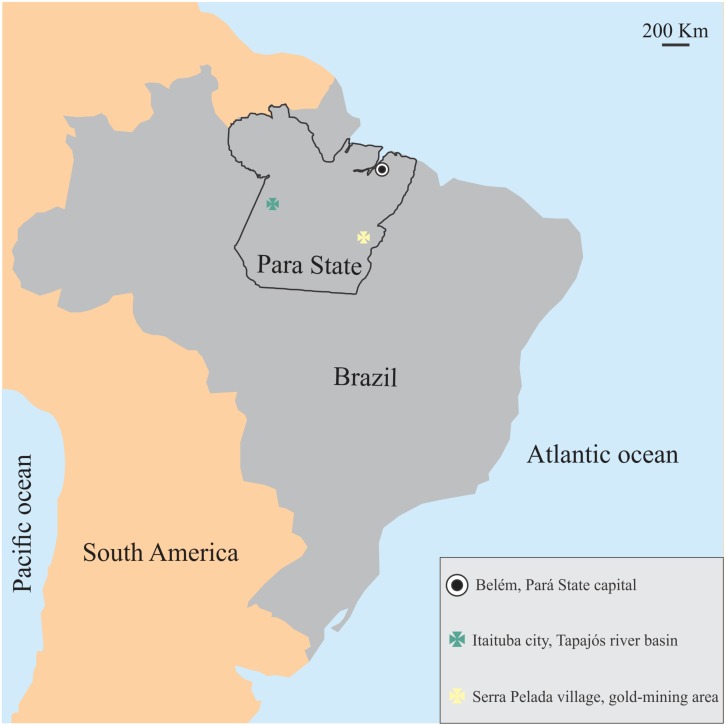
Location of the mercury-exposed communities studied in the present investigation. Both communities shared similar geographical, economical, environmental conditions, but differed in mercury exposure.

The sample is comprised of 44 total subjects, forming two groups according to the type of mercury exposure: 10 riverines (all males, 44.8 ± 14.3 year old) environmentally exposed to mercury by fish eating who lived in the Tapajós River basin, Pará, Brazil; and 34 gold-miners (all males, 46.2 ± 8.6 year old) occupationally exposed to mercury vapor from Serra Pelada mining, Pará, Brazil. Figure 1 shows the location of the communities studied in the present investigation.

The riverside communities from the Tapajós River basin are located near Itaituba a medium sized port city in the West of Pará State, Brazil. These villages are located around regions historically known as mineral extraction regions that used mercury in the artisanal process of gold extraction, an important source of environmental contamination. Moreover, there is important deforestation around the city of Itaituba that should be considered as source of mercury in the region. No participant from Tapajós river communities inhaled mercury vapor, and mercury exposure was mainly dependent on fish consumption. Serra Pelada village is located near the small city called Curionópolis, in the Southeast of the state of Pará, Brazil. It is a region that constituted one of most important sites of gold extraction mining in the 1980’s and was still active at the moment of the study. The workers involved in this activity were strongly exposed to mercury vapor originated from amalgamation of gold in the metal extraction process.

These communities shared the same culture, language, and socio-environmental conditions and they are in the same state of Brazil. They also had little education, poor sanitation, and low-paying jobs. All the participants from both communities reported a non-systematic smoking habit or even alcoholic beverage drinking habit.

On our arrival to each village, the invitation procedure to join this study was similar: a meeting was called by the local community health nursing aides to explain the study purposes to the villagers and to invite them to participate. The study took place at the Community Public Health Post and a questionnaire including socio-demographic information, smoking and drinking habits, fish weekly intake, and medical and work history was given by interview; the entire procedure took approximately 1 h. We tested a different number of participants for each visual test, depending on their availability.

### Hair Mercury Exposure Quantification

Hair samples were analyzed using cold vapor atomic absorption spectrometry (Mercury Analyzer HG-201, Sanso Seisakusho, Tokyo, Japan) according to a previously published protocol (Suzuki et al., 2004). Analytical quality control was warranted by International Atomic Energy Agency certification (IAEA-085) and the measurements were performed in duplicate. All the results were expressed as μg Hg per g hair (μg/g).

Human hair material (IAEA-086) was the reference to validate the mercury concentration.

### Visual Tests

All the subjects had visual acuity of 20/40 or better in both eyes. We chose the eye with better visual acuity to be tested.

#### Visual Perimetry

All tests were carried out monocularly. The visual perimetry was estimated using the Förster perimeter. Foster perimeter is a broad semicircular arc that can be rotated manually on its axis. There is a fixation point at the arc center and a white dot at the arc that can be moved along the arc. The patient was instructed to fixate on the center of the equipment. The experimenter moved the white dot from the border of the arc toward to the center. The patient was instructed to inform the moment that the white dot is detect. The test is performed in different rotating angles of the arc, and the experimenter recorded the angle at the arc which was detected the white dot. At the end of the experiment, we quantified the perimetric area.

#### Hue Ordering Test

The hue ordering test was a home-made computerized version of the Farnsworth–Munsell 100 hue test (Bento-Torres et al., 2016). It consisted of 85 circular stimuli (1° of visual angle, mean luminance of 41.75 cd/m^2^) of different hues and same saturation. Four sets of caps were shown separately in the same order of the conventional test. Initially, the correct sequence of the caps was presented to the participant, followed by its disarrangement. The participants were instructed to reorder as close as possible in the original hue sequence. We measured the arrangement errors for each cap position and the total error score (TES) such as done in Bento-Torres et al. (2016) and Feitosa-Santana et al. (2018). The error calculation considered that the caps had values from 1 to 85. After the participant completed the hue ordering task, we calculated the partial error score (PES) for each cap (Eq. 1), as the sum of the absolute difference between the cap value in the position *i* and in the neighbor positions *i*+1 and *i*-1. For each cap, the correct ordering resulted in a PES value of 2.

(1) $$\text{PES}=\left|n_{i}-n_{i-1}\right|+\left|n_{i}-n_{i+1}\right|$$

Total error score was considered as the sum of all 85 PES minus 170. The perfect performance resulted in a TES of 0. The hue ordering results were transformed to square root values.

#### Statistics

The hue ordering score of the exposed groups were compared to the database of an age-matched control group that lived in an urban region without relevant contact to Hg contamination sources (*n* = 41 male volunteers). All the controls had no history of systemic or neurological diseases that influenced the visual function and had normal or corrected to 20/20 visual acuity. We considered the perimetric area of 57.07 cm^2^ as reference value for normative results of the visual perimetry as informed by the device manufacturer (American Optical Company, United States).

We compared the hair mercury concentration and the visual perimetric area obtained from each group using the *t*-test with Welch’s correction. We used *G*-test to compare the fish weekly intake of the groups. We used Welch one-way ANOVA followed by Tukey test *post hoc* to compare the color vision outcomes among the control, riverines and gold-miners. We evaluated the linear correlation between both visual outcomes from each community using the Pearson product-moment correlation. For all comparisons, we considered the significance level of 0.05.

## Results

### Mercury Exposure

**FIGURE 2:**
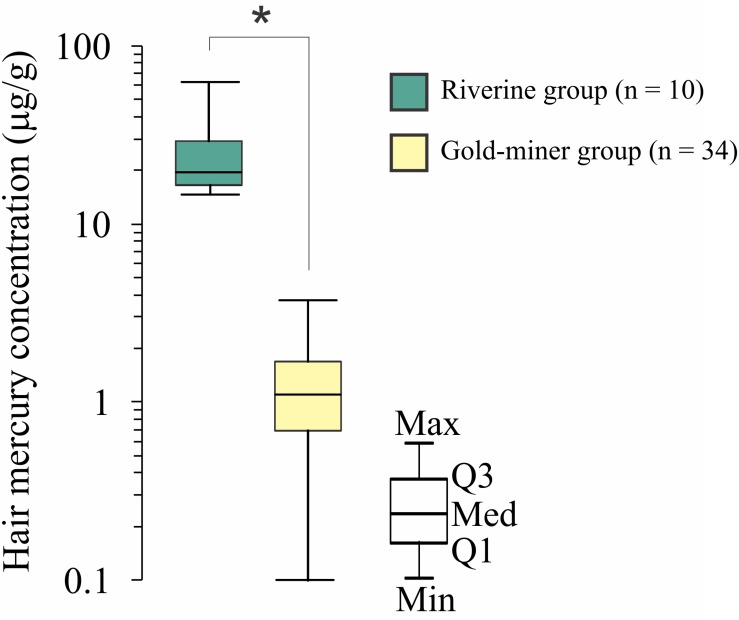
Hair mercury concentration comparison between riverines and gold-miners. Riverines has significant higher hair mercury concentration than the gold-miners. ^∗^Significant difference, *p* < 0.05. Box-plots are composed by first (Q1), second (Q2), and third (Q3) quartiles, and whiskers represent the maximum (Max) and minimum (Min) values.

The Table 1 shows the age and fish weekly consumption from both groups. Both groups were age matched. The riverines had a higher fish weekly intake compared to the gold-miners. All the gold-miners used to have fish less than twice in a week, while the riverines have more than two meals including fish during the week. Figure 2 shows the mercury exposure of each community. We observed that the riverine population had higher mercury concentration compared to the gold-miners [*t*(9.02) = 5.159, *p* = 0.0006].

**TABLE 1:** Demographic characteristics and fish weekly intake of both communities.

**Variable**	**Riverines**	**Gold-miners**	***p*-value**
*Age (year old)*	40.86 ± 13.2	45.9 ± 8.5	0.11^a^
*Fish weekly intake*			
<2	–	34	0.001^b^
2–4	4	–	
>4	6	–	

*^*a*^*t*-test; ^*b*^*G*-test.*

### Visual Function Evaluation

**FIGURE 3:**
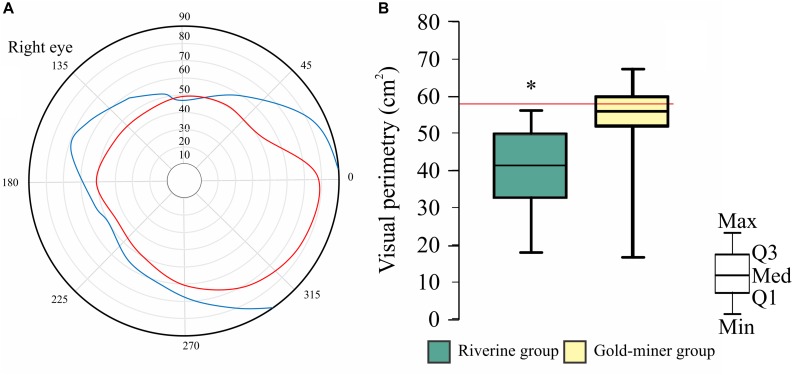
Visual perimetric results. **(A)** Visual perimetry of a standard observer (blue line) and of a mercury exposed observer (red line). **(B)** Visual perimetric area distribution of the riverine (green box-plot) and gold-miner (yellow box-plot) group. ^∗^Significant difference, *p* < 0.05. Box-plots are composed by first (Q1), second (Q2), and third (Q3) quartiles, and whiskers represent the maximum (Max) and minimum (Min) values.

Figure 3 shows an example of the perimetry obtained from the right eye of a standard observer (in blue) and a mercury exposed subject (in red). All riverine subjects had a perimetric area smaller than the reference value, while 61.8% of the gold-miner group (21/34 subjects) were below the reference level. We found that the riverine population had smaller perimetric area compared to the gold-miners results [*t*(31.57) = 3.613, *p* = 0.001].

**FIGURE 4:**
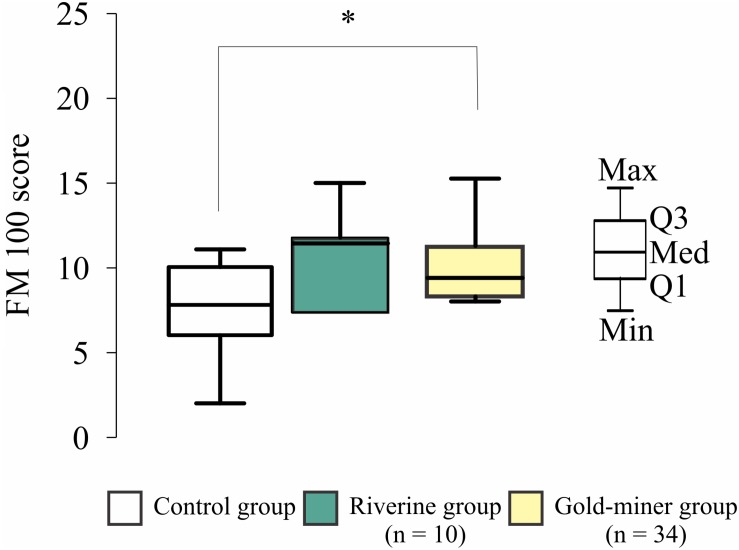
Hue ordering results. Distribution of control (white box-plot), riverine (green box-plot), and gold-miner (yellow box-plot) groups. Both mercury exposed groups had higher error scores than the controls (^∗^*p* < 0.05). Box-plots are composed by first (Q1), second (Q2), and third (Q3) quartiles, and whiskers represent the maximum (Max) and minimum (Min) values.

Figure 4 shows the comparison among control, riverines and gold-miners results obtained in the hue ordering test. Gold-miners had larger amounts of error than the controls [One-way ANOVA, *F*(2,10.69) = 5.764, *p* = 0.02], but there was no difference between the results obtained from the two mercury-exposed groups and between riverines and controls. Although, we have observed no difference between controls and riverines, the riverines had error closer to the gold-miners performance than to the controls.

**FIGURE 5:**
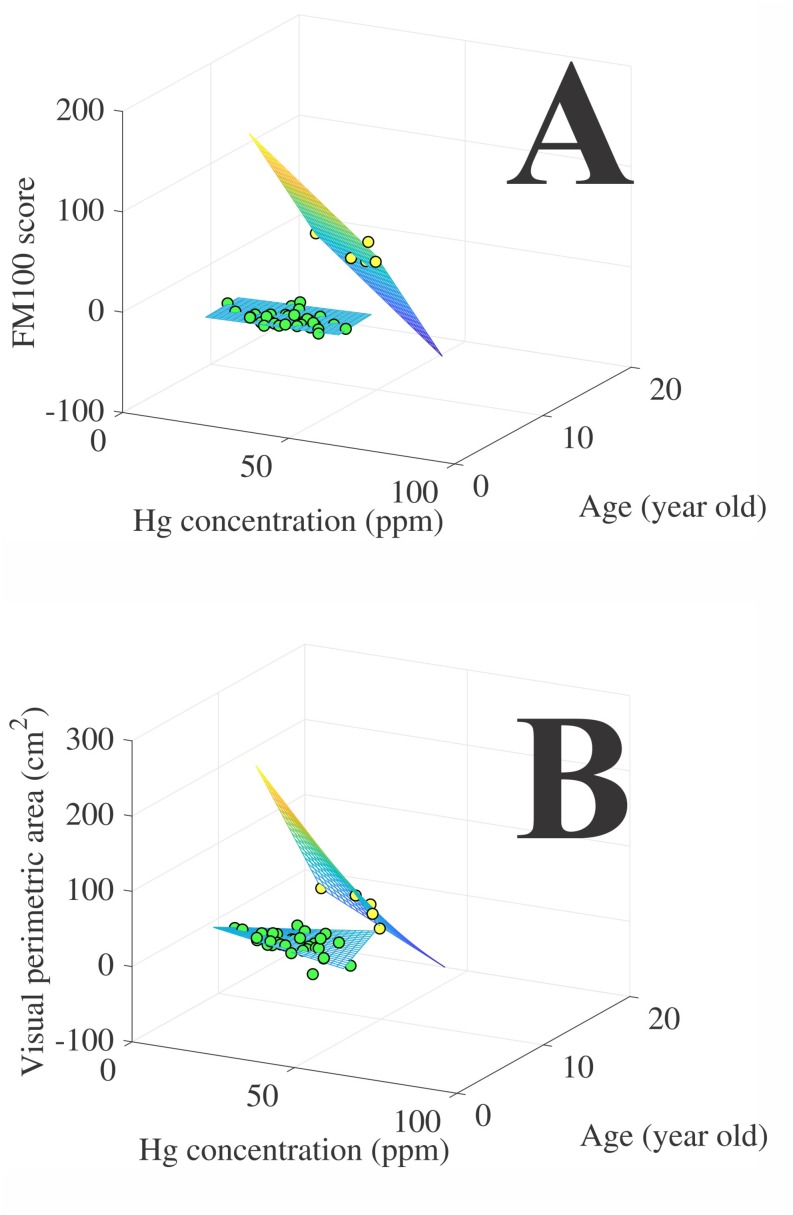
Multiple regression between the visual outcomes (**A**, hue ordering test performance; **B**, visual perimetric area) and the independent variables (age and hair mercury concentration). Green circles represent the riverines data points (*n* = 5) and yellow circles represent gold-miners data points (*n* = 34). The grid represents the best three-dimensional model that fitted the visual outcomes to the independent variables.

The results of the multiple linear correlation among visual outcomes, age and hair mercury concentration are shown in the Table 2. We observed that both mercury exposed groups had no significant multiple correlation, but only the riverines had significant partial correlation between mercury exposure and visual outcome (visual perimetry). The visual outcomes of the gold-miners was not significantly associated to the hair mercury concentration and age. Figure 5 shows the multiple correlation between the visual outcomes and the independent variables of age and hair mercury concentration.

**TABLE 2:** Multiple linear regression results.

**Dependent**	**Partial r (Hg concentration)/**	**Partial r (age)/**	**R^2^/*p*-value**
**variable**	***p*-value**	***p*-value**	
*Riverines*			
Visual perimetry	0.56/0.04	0.04/0.87	0.46/0.1
Color vision	−0.08/0.33	−0.09/0.47	0.82/0.06
*Gold-miners*			
Visual perimetry	0.06/0.97	−0.25/0.16	0.06/0.12
Color vision	0.53/0.21	0.08/0.09	0.05/0.16

*R^2^ = coefficient of multiple determination, r = coefficient of regression.*

We correlated the visual outcomes from each group. We found a significant linear correlation between the visual evaluation results obtained from the riverine population (*p* = 0.004), that was described by a negative correlation with linear coefficient of 0.97. For the gold-miners, no significant correlation was observed for their visual evaluation (*r* = -0.24, *p* = 1755).

## Discussion

The present investigation was the first comparison of visual performance between different groups of mercury-exposed subjects living in the same region but with different types of exposure. Our main result was that both mercury exposed groups had visual deficits, but riverines showed greater visual impairment than gold miners.

The association between the mercury exposure and the severity of the decreased visual function is indicated by three findings: (i) the riverines had higher exposure to the mercury than gold-miners; (ii) riverines had worse performance for the visual perimetry than the gold-miners, but gold-miners had smaller performance in the color vision test than riverines; (iii) riverines’ visual outcomes were highly correlated, the poor color vision, the smaller perimetric area, but the same was not found for the gold-miners what can be indicative of lower influence of the mercury exposure on the visual system in this participants.

The comparison between the mercury exposure suffering by riverines and gold-miners should be made carefully, because both groups have distinct types of mercury exposure and the metabolism of the metal in their bodies is completely different. We observed that the riverines had higher hair mercury exposure than the gold-miners. Our results showed that the riverines had higher fish weekly intake than the gold-miners. The fish intake is positively associated to the hair mercury concentration (Passos et al., 2008).

The visual function has been used as biomarker of the mercury exposure. No specific mechanism of visual loss has been fully described, but probably involves alterations since from the optical apparatus of the eye up to visual cortex. Mercury has a cataractogenic potential because it can induce aggregation of human lens proteins (Domínguez-Calva et al., 2018), electroretinographic and cortical recordings in mercury exposed humans suggest impairment of the retinal and cortical activity (Ventura et al., 2004, 2005; Saldana et al., 2006; da Costa et al., 2008; Yorifuji et al., 2013). The influence of mercury in the visual perception has been investigated by many psychophysical tests for hue ordering test, chromatic discrimination, visual acuity, and visual field sensitivity (Cavalleri et al., 1995; Cavalleri and Gobba, 1998; Rodrigues et al., 2007; Fillion et al., 2011, 2013; Dos Santos Freitas et al., 2018; Feitosa-Santana et al., 2018).

Other investigations evaluated the visual field of subjects exposed to mercury vapor (Barboni et al., 2008). Exposed subjects were factory workers that inhaled the mercury vapor during the manufacturing of fluorescent lamps. A constriction of the visual field was observed in these subjects. In the present study, we observed that the gold-miners, despite being associated with mercury vapor exposure, had normal visual fields. This difference could be explained by the factory workers that had a constant exposure to mercury vapor in the work environment (constant exposure), while the gold-miners only had exposure during the process of gold extraction (intermittent exposure). Additionally, the present study and Barboni et al. (2008) differed in the method to evaluate the visual field. In the factory workers evaluation, static Humphrey visual perimetry – that measured the luminance threshold for different locations of the visual field – was used, while we used the dynamic perimetry to identify the boundaries of the visual field. The test we chose is less sensitive than the static visual field analyzer, but it is portable, which makes it possible to take it for field work in the Amazonian villages.

Previously, other investigations have shown color vision loss in fish consuming riverine populations (Rodrigues et al., 2007; Dos Santos Freitas et al., 2018; Feitosa-Santana et al., 2018) in the Amazon region. Most of them have used hue ordering tests to evaluate the color vision of the exposure subjects (Dos Santos Freitas et al., 2018; Feitosa-Santana et al., 2018). We confirmed the previous findings showing that color vision is altered in subjects with no history of mercury vapor exposure, but have fish as their main source of proteins. We also confirmed that color vision is altered in mercury vapor exposure subjects, as observed in the factory workers. Our new contribution about color vision in Amazonian populations is that there was no difference in the color vision deficits shown by both groups of mercury exposed subjects.

We cannot assert that all the visual disturbances that we found are caused by mercury, but as both communities have similar socioeconomical profile (low paying-jobs, poor sanitation, infectious diseases), age, environmental condition (sunlight exposure and climate), we consider that the difference of mercury exposure is associated to the visual differences between the communities. The inhalation of the mercury vapor is the primary route of entry into the body for inorganic mercury. Its absorption is fast by diffusion in the lungs and the half-life estimated in the body is about 60 days. Its excretion is mainly by the feces and urine elimination (Sandborgh-Englund et al., 1998; Park and Zheng, 2012). In the body, the elemental mercury is converted to an oxidized form, which does not effectively cross the blood-brain barrier (Friberg and Mottet, 1989). Methylmercury is separated from the food by the gastric acid and is absorbed in the duodenum. It has high affinity with lipophilic tissues and easily crosses the blood-brain barrier and accumulates in the nervous tissue (Lee et al., 2006, Korean Food and Drug Administration, 2007).

We concluded that the Amazonian population exposed to different mercury forms showed similar visual deficits, with greater impairment in riverine communities. Our findings indicated that these different populations need specific health and education programs to become conscious of the dangers of exposure to mercury. People could very well be aware of the dangers of mercury, but avoiding it could be a greater challenge as fish is a dietary mainstay for the riverine population and gold extraction is one of the few economical activities in the Serra Pelada region. Larger scale interventions should aim at reducing mercury at the source, and the delivery of health services should also be improved.

## Ethics Statement

All procedures were evaluated and approved by the Ethics Committee in Research in Humans of the Tropical Medicine Center of the Federal University of Pará (Protocol #021/2009-CEP-NMT/UFPA).

## Author Contributions

All authors contributed to the conception of the work, drafting and revising the manuscript, approved the final version, and agree to be accountable for all aspects of the work. LS, MP, and DV designed the experiments. MC, EL, and AR carried out the psychophysical experiments. GS and EL analyzed the data. EL, GS, and DV wrote the components of the manuscript.

## Conflict of Interest

The authors declare that the research was conducted in the absence of any commercial or financial relationships that could be construed as a potential conflict of interest.

## References

[B1] Akagi H. Naganuma N. (2000). Human exposure to mercury and the accumulation of methylmercury that is associated with gold mining in the Amazon Basin, Brazil. *J. Health Sci.* 46 323–328. 10.1248/jhs.46.323

[B2] Amin-Zaki L. Elhassani S. Mohammed M. A. Clarkson T. W. Doherty R. A. Greenwood M. R. (1976). Giovanoli-Jakubczak, T. Perinatal methylmercury poisoning in Iraq. *Am. J. Dis. Child.* 130 1070–1076. 973609 10.1001/archpedi.1976.02120110032004

[B3] Barboni M. T. da Costa M. F. Moura A. L. Feitosa-Santana C. Gualtieri M. Lago M. (2008). Visual field losses in workers exposed to mercury vapor. *Environ. Res.* 107 124–131. 10.1016/j.envres.2007.07.004 17719027

[B4] Bento-Torres N. V. Rodrigues A. R. Côrtes M. I. Bonci D. M. Ventura D. F. Silveira L. C. (2016). Psychophysical evaluation of congenital colour vision deficiency: discrimination between protans and deutans using Mollon-Reffin’s ellipses and the Farnsworth–Munsell 100-hue test. *PLoS One* 11:e0152214. 10.1371/journal.pone.0152214 27101124 PMC4839569

[B5] Cavalleri A. Belotti L. Gobba F. Luzzana G. Rosa P. Seghizzi P. (1995). Colour vision loss in workers exposed to elemental mercury vapour. *Toxicol. Lett.* 77 351–356. 10.1016/0378-4274(95)03317-3 7618161

[B6] Cavalleri A. Gobba F. (1998). Reversible color vision loss in occupational exposure to metallic mercury. *Environ. Res.* 77 173–177. 10.1006/enrs.1997.3814 9600811

[B7] da Costa G. M. dos Anjos L. M. Souza G. S. Gomes B. D. Saito C. A. Pinheiro Mda C. (2008). Mercury toxicity in Amazon gold miners: visual dysfunction assessed by retinal and cortical electrophysiology. *Environ. Res.* 107 98–107. 10.1016/j.envres.2007.08.004 17889848

[B8] Domínguez-Calva J. A. Pérez-Vázquez M. L. Serebryany E. King J. A. (2018). Mercury-induced aggregation of human lens γ-crystallins reveals a potential role in cataract disease. *J. Biol. Inorg. Chem.* 23 1105–1118. 10.1007/s00775-018-1607-z 30167892

[B9] Dos Santos Freitas J. da Costa Brito Lacerda E. M. da Silva Martins I. C. V. Rodrigues D. Jr. Bonci D. M. O. Cortes M. I. T. (2018). Cross-sectional study to assess the association of color vision with mercury hair concentration in children from Brazilian Amazonian riverine communities. *Neurotoxicology* 65 60–67. 10.1016/j.neuro.2018.02.006 29428869

[B10] Ekino S. Susa M. Ninomiya T. Imamura K. Kitamura T. (2007). Minamata disease revisited: an update on the acute and chronic manifestations of methyl mercury poisoning. *J. Neurol. Sci.* 262 131–144. 10.1016/j.jns.2007.06.036 17681548

[B11] Feitosa-Santana C. Barboni M. T. Oiwa N. N. Paramei G. V. Simões A. L. Da Costa M. F. (2008). Irreversible color vision losses in patients with chronic mercury vapor intoxication. *Vis. Neurosci.* 25 487–491. 10.1017/S0952523808080590 18598423

[B12] Feitosa-Santana C. Costa M. F. Lago M. Ventura D. F. (2007). Long-term loss of color vision after exposure to mercury vapor. *Braz. J. Med. Biol. Res.* 40 409–414. 10.1590/s0100-879x2007000300017 17334539

[B13] Feitosa-Santana C. Souza G. D. S. Sirius E. V. P. Rodrigues A. R. Cortes M. I. T. Silveira L. C. L. (2018). Color vision impairment with low-level methylmercury exposure of an Amazonian population - Brazil. *Neurotoxicology.* 66 179–184. 10.1016/j.neuro.2018.01.010 29432854

[B14] Figueiredo B. R. De Campos A. B. Da Silva R. Hoffman N. C. (2018). Mercury sink in Amazon rainforest: soil geochemical data from Tapajos National Forest, Brazil. *Environ. Earth Sci.* 77:296.

[B15] Fillion M. Lemire M. Philibert A. Frenette B. Weiler H. A. Deguire J. R. (2013). Toxic risks and nutritional benefits of traditional diet on near visual contrast sensitivity and color vision in the Brazilian Amazon. *Neurotoxicology* 37 173–181. 10.1016/j.neuro.2013.04.010 23680050

[B16] Fillion M. Philibert A. Mertens F. Lemire M. Passos C. J. Frenette B. (2011). Neurotoxic sequelae of mercury exposure: an intervention and follow-up study in the Brazilian Amazon. *Ecohealth* 8 210–222. 10.1007/s10393-011-0710-1 22160443

[B17] Friberg L. Mottet N. K. (1989). Accumulation of methylmercury and inorganic mercury in the brain. *Biol. Trace Elem. Res.* 21 201–206. 10.1007/bf02917253 2484587

[B18] Kershaw T. G. Clarkson T. W. Dhahir P. H. (1980). The relationship between blood levels and dose of methylmercury in man. *Arch. Environ. Health* 35 28–36. 10.1080/00039896.1980.10545720 7189107

[B19] Korean Food and Drug Administration (2007). *Hazardous Substances-21 Series: What is Methylmercury in Food?* Seoul: Korea Food and Drug Administration, 1–68. (Korean).

[B20] Lacerda L. D. (1997). Contaminação por mercúrio no Brasil: fontes industriais vs garimpo de ouro. *Química Nova* 20 196–199. 10.1590/s0100-40421997000200012

[B21] Lebel J. Mergler D. Branches F. Lucotte M. Amorim M. Larribe F. (1998). Neurotoxic effects of low-level methylmercury contamination in the Amazonian Basin. *Environ. Res.* 79 20–32. 10.1006/enrs.1998.3846 9756677

[B22] Lebel J. Mergler D. Lucotte M. Amorim M. Dolbec J. Miranda D. (1996). Evidence of early nervous system dysfunction in Amazonian populations exposed to low-levels of methylmercury. *Neurotoxicology* 17 157–167. 8784826

[B23] Lee C. Cho S. D. Chang D. S. Shin D. H. Oh D. H. Whang I. (2006). Food safety guidelines for consumer. *Safe Food* 1 31–43.

[B24] Malm O. Branches F. J. Akagi H. Castro M. B. Pfeiffer W. C. Harada M. (1995). Mercury and methylmercury in fish and human hair from the Tapajós river basin, Brazil. *Sci. Total Environ.* 175 141–150. 10.1016/0048-9697(95)04910-x 8560242

[B25] Magarelli G. Fostier A. H. (2005). Influence of deforestation on the mercury air/soil exchange in the Negro River Basin. *Amazon. Atmosph. Environ.* 39 7518–7528. 10.1016/j.atmosenv.2005.07.067

[B26] Park J. D. Zheng W. (2012). Human exposure and health effects of inorganic and elemental mercury. *J. Prev. Med. Public Health* 45 344–352. 10.3961/jpmph.2012.45.6.344 23230464 PMC3514464

[B27] Passos C. J. Da Silva D. S. Lemire M. Fillion M. Guimarães J. R. Lucotte M. (2008). Daily mercury intake in fish-eating populations in the Brazilian Amazon. *J. Expo. Sci. Environ. Epidemiol.* 18 76–87. 10.1038/sj.jes.7500599 17805232

[B28] Passos C. J. Mergler D. (2008). Human mercury exposure and adverse health effects in the Amazon: a review. *Cadernos de Saude Publica* 24(Suppl. 4), s503–s520. 10.1590/s0102-311x2008001600004 18797727

[B29] Rodrigues A. R. Souza C. R. Braga A. M. Rodrigues P. S. Silveira A. T. Damin E. T. (2007). Mercury toxicity in the Amazon: contrast sensitivity and color discrimination of subjects exposed to mercury. *Braz. J. Med. Biol. Res.* 40 415–424. 10.1590/s0100-879x2007000300018 17334540

[B30] Roulet M. Lucotte M. Canuel R. Rheault I. Tran S. De Freitos Gob Y. G. (1998). Distribution and partition of total mercury in waters of the Tapajós River Basin, Brazilian Amazon. *Sci. Total Environ.* 213 203–211.

[B31] Saldana M. Collins C. E. Gale R. Backhouse O. (2006). Diet-related mercury poisoning resulting in visual loss. *Br. J. Ophthalmol.* 90 1432–1434. 10.1136/bjo.2006.094821 17057175 PMC1857490

[B32] Sandborgh-Englund G. Elinder C. G. Langworth S. Schütz A. Ekstrand J. (1998). Mercury in biological fluids after amalgam removal. *J. Dent. Res.* 77 615–624. 10.1177/00220345980770041501 9539465

[B33] Suzuki T. Akagi H. Arimura K. Ando T. Sakamoyo M. Satoh H. (2004). *Mercury Analysis Manual.* Tokyo: Ministry of Environment.

[B34] Takeuchi T. (1968). *Pathology of Minamata Disease.* Kumamoto: Kumamoto University.

[B35] Takeuchi T. (1977). “Neuropathology of Minamata disease in Kumamoto: especially at the chronic stage,” in *Neurotoxicology*, eds Roisin L. Shiaki H. Greevic N., (New York: Raven Press), 235–246.

[B36] Ventura D. F. Costa M. T. Costa M. F. Berezovsky A. Salomão S. R. Simões A. L. (2004). Multifocal and full-field electroretinogram changes associated with color-vision loss in mercury vapor exposure. *Vis. Neurosci.* 21 421–429. 10.1017/s0952523804213372 15518224

[B37] Ventura D. F. Simões A. L. Tomaz S. Costa M. F. Lago M. Costa M. T. (2005). Colour vision and contrast sensitivity losses of mercury intoxicated industry workers in Brazil. *Environ. Toxicol. Pharmacol.* 19 523–529. 10.1016/j.etap.2004.12.016 21783522

[B38] WHO, International Program on Chemical Safety, (1989). *Environmental Health Criteria 86: Mercury-Environmental Aspects.* Geneva: World Health Organization.

[B39] Yorifuji T. Murata K. Bjerve K. S. Choi A. L. Weihe P. Grandjeane P. (2013). Visual evoked potentials in children prenatally exposed to methylmercury. *Neurotoxicology* 37 15–18. 10.1016/j.neuro.2013.03.009 23548974 PMC3696435

